# Anti-cancer effect of *Cordyceps militaris* in human colorectal carcinoma RKO cells via cell cycle arrest and mitochondrial apoptosis

**DOI:** 10.1186/s40199-015-0117-6

**Published:** 2015-07-04

**Authors:** Hwan Hee Lee, Seulki Lee, Kanghyo Lee, Yu Su Shin, Hyojeung Kang, Hyosun Cho

**Affiliations:** College of Pharmacy, Duksung Women’s University, Seoul, 132-714 Republic of Korea; Innovative Drug Center, Duksung Women’s University, Seoul, 132-714 Republic of Korea; Mushroom Research Division, National Institute of Horticultural and Herbal Science, Rural Development Administration, Eumseong, 369-873 Republic of Korea; Department of Medicinal Crop Research, National Institute of Horticultural and Herbal Science, Rural Development Administration, Eumseong, 369-873 Republic of Korea; College of Pharmacy, Research Institute of Pharmaceutical Sciences and Institute for Microorganisms, Kyungpook National University, Daegu, 702-701 Republic of Korea

**Keywords:** *Cordyceps militaris*, Human colorectal carcinoma, p53, Mitochondrial-mediated apoptosis

## Abstract

**Background:**

*Cordyceps militaris* has been used as a traditional medicine in Asian countries for a long time. Different types of Cordyceps extract were reported to have various pharmacological activities including an anti-cancer effect. We investigated the inhibitory effect of *Cordyceps militaris* ethanol extract on a human colorectal cancer-derived cell line, RKO.

**Methods:**

RKO cells were treated with various concentrations of nucleosides-enriched ethanol extract of *Cordyceps militaris* for 48 h and cytotoxicity was measured using a CCK-8 assay. Then, xenograft Balb/c nude mice were injected with RKO cells and subsequently orally administered with ethanol extract of *Cordyceps militaris* every day for 3 weeks to examine the inhibitory effect on tumor growth. Lastly, the effect of *Cordyceps militaris* on cell cycle as well as apoptosis was measured using flow cytometry. Also, the expression of p53, caspase 9, cleaved caspase-3, cleaved PARP, Bim, Bax, Bak, and Bad were detected using western blot assay.

**Results:**

RKO cells were highly susceptible to the ethanol extract of *Cordyceps militaris* (CME) and the growth of RKO cells-derived tumor was significantly delayed by the treatment of *Cordyceps militaris. Cordyceps militaris* induced cell cycle arrest in G2/M phase (untreated; 20.5 %, CME 100 μg/ml; 61.67 %, CME 300 μg/ml; 66.33 %) and increased early apoptosis (untreated; 1.01 %, CME 100 μg/ml; 8.48 %, CME 300 μg/ml; 18.07 %). The expression of p53, cleaved caspase 9, cleaved caspase-3, cleaved PARP, Bim, Bak, and Bad were upregulated by the treatment of *Cordyceps militaris*.

**Conclusion:**

Ethanol extract of *Cordyceps militaris* was highly cytotoxic to human colorectal carcinoma RKO cells and inhibited the growth of tumor in xenograft model. The anti-tumor effect of *Cordyceps militaris* was associated with an induction of cell cycle arrest and mitochondrial-mediated apoptosis.

## Background

A large number of edible mushrooms have been shown to possess various biological activities [1–5]. Edible mushrooms from Pleurotus species showed the antioxidative, antimicrobial and anrifungal properties when they were cultivated on banana agrowastes [1, 2]. Antimetastatic activity of *Hericium erinaceus* edible mushroom extracts was reported using murine colon carcinoma cells [3]. Antioxidant and cytotoxic activities of ethanolic extracts of *Phellinus Quél* were recently reported [4]. An extract of *Agaricus blazei Murill* was shown to stimulate immunocytes and regulate immune response in leukemia mice [5]. *Cordyceps* mushrooms also have received extensive attention owing to their potent pharmacological activities. *Cordyceps sinensis* and *Cordyceps militaris* are representative species for their medicinal uses in China and Korea. Both of them are fungi on the larvae of caterpillars of moths and traditional medicine involves both mushroom fruitbody and parasitized larvae. A couple of well-known active ingredients in these mushrooms include cordycepin, cordycepic acid, sterols (ergosterol), nucleosides, and polysaccharides [6, 7]. *Cordyceps militaris* is a traditional Chinese medicine*,* which has been cultured successfully and has been shown to have a higher content of cordycepin and cordycepic acid than *Cordyceps sinensis* [8]. Diffferent types of extracts of *Cordyceps militaris* have been reported to exert immunomodulatory, anti-inflammatory, anti-microbial and antitumor effects although the primary pharmacological activity is a little bit different depending on the main ingredients of extract [7–10]. Recently, we found that ethanol extract of *Cordyceps militaris*, which has a couple of nucleoside analogs as the main components, had an anti-influenza effect in a DBA2 mouse model [11].

Beside the routine methods of surgery, radiotherapy and chemotherapy, traditional herb medicine is one of the major complementary and alternative medicines for treating various malignant diseases, including colorectal cancer [12–14].

Colorectal cancer is a serious health problem that has progressively increased to be one of the most common cancers in Asian countries [15]. *Cordyceps* extract has been reported to have a potent cytotoxic effect on various human cancer cells, including human lung carcinoma cells [16, 17]. However, the anti-tumor effect of *Cordyceps* extract on human colorectal cancer cells was not precisely examined *in vivo* model. Xenograft mouse model is immunocompromised animal, which could be implanted with the human tumor cells either under the skin or into any organ and does not reject the tumor. Therefore, it has been an indispensable model system for the preclinical screen and the development of novel anti-cancer agents.

p53 is known to play a critical role in the induction of cell apoptosis in response to DNA damage [18]. A typical pro-apoptotic molecule activated by p53 is Bax, a member of the Bcl-2 family [19]. The Bcl-2 family of proteins includes both pro-apoptotic members (Bak, Bax, and Bad) as well as anti-apoptotic members (Bcl-2, Bcl-xL, and Bcl-w). Mitochondria-dependent cell apoptosis is regulated mainly by the ratio of expression of Bcl-2 family proteins [20]. The activation of Bax results in the release of cytochrome c into the cytosol, which leads to the activation of caspases 9 and 3, effector caspases, in the mitochondrial pathway of cell apoptosis [21, 22].

In the present study, we investigated the cytotoxic effect of *Cordyceps militaris* ethanol extract on human colorectal carcinoma RKO cells and evaluated the anti-cancer effect of *Cordyceps militaris* in mice bearing RKO cell-derived tumors. Subsequently, the underlying mechanisms, which mediate the anti-cancer effect of *Cordyceps militaris* on human colorectal cancer, were precisely examined. We found that the anti-cancer effect of nucleosides-enriched ethanol extract of *Cordyceps militaris* was highly associated with the increased expression of p53, Bax, Bim, Bak, Bad, cleaved-caspases 9 and 3, and PARP.

## Material and methods

### Specimen preparation

The fungus strain *Cordyceps militaris* was from Dong-Chong-Xia-Cao Culture Collection (Mushtech, Hoengseong, Kangwondo, Reupblic of Korea). Fresh fruiting bodies or mycelia of *Cordyceps militaris* were extracted with 50 % ethanol at room temperature for 3 days. The extracts were filtered, concentrated, sterilized and dried as previously described [23]. The major compounds of extract are cordycepin, adenosine, urasil and guanosine, which structurally belong to nucleoside family (provided by Dong-a Pharm. Co., LTD, Yongin, Republic of Korea). Extracts of specimens were diluted with distilled water for RKO cell treatment as well as for oral administration in the mouse experiment.

### Cell culture and treatment

Human colorectal carcinoma (RKO) cells were obtained from ATCC and cultured in Dulbecco’s Modified Eagle’s medium (DMEM, Gibco, USA) supplemented with 10 % heat-inactivated fetal bovine serum (FBS, Hyclone, USA), 100 U/ml penicillin and streptomycin (Gibco, USA) at 37 °C in a humidified atmosphere with 5 % CO_2_.

### Cell cytotoxicity

Cytotoxicity of ethanol extract of *Cordyceps militaris* on human colorectal carcinoma (RKO) cells was assessed with the Cell Counting Kit-8 (CCK-8, Dojindo, Japan) as previously described [24]. Briefly, cells were seeded in 96-well plates at a density of 3 × 103 cells/well and treated with different concentrations of ethanol extract of *Cordyceps militaris* (0, 25, 50, 100, 250, 500, and 1000 μg/ml) for 24 h or 48 h. CCK-8 solution was added to each well and incubated for another 3 h. The absorbance was measured using a microplate reader (BMG Labtech, Germany) at 450 nm.

### Cell morphology

Cells were seeded at a density of 4 × 104 cells/well in 6-well plates. After incubation for 24 h, the cells were treated with different concentrations of ethanol extract of *Cordyceps militaris* (0, 100, and 300 μg/ml) and incubated for 24 h. The cells were observed by light microscopy (×200) (Nikon eclipse TS100, Japan).

### Cell cycle analysis

Cells were seeded at a density of 1 × 106 cells/well in 6-well plates, and treated with different concentrations of ethanol extract of *Cordyceps militaris* (0, 100, and 300 μg/ml) for 24 h. Cells were harvested by trypsinization and fixed with 70 % ethanol at 4 °C overnight. The next day, cells were resuspended in PBS buffer containing 0.2 mg/ml RNase A (Qiagen, USA) and incubated for 1 h at 37 °C. Cells were then stained with propidium iodide for 30 min in the dark. Stained cells were analyzed using a flow cytometer (EasyCyte guava, Merck Millipore).

### Annexin V-FITC and propidium iodide assay

Cell apoptosis was analyzed using the Annexin V-FITC Apoptosis Detection Kit (Roche, USA) as previously described [24]. Briefly, RKO cells were plated in 6-well plates at a density of 1 × 106 cells/well and incubated for 24 h. Cells were treated with different concentrations (0, 100, and 300 μg/ml) of ethanol extract of *Cordyceps militaris* for 24 h or 48 h and then Annexin V and PI solution were added. Cells were analyzed by flow cytometry (EasyCyte guava, Merck Millipore).

### Western blot analysis

Western blot assays were performed as previously described with modification [24]. Briefly, RKO cells were treated with ethanol extract of *Cordyceps militaris* (100 and 300 μg/ml) for 48 h. Protein from cell lysates was measured using the Bradford assay, separated by electrophoresis, and transferred onto nitrocellulose membranes. Membranes were subsequently incubated with 1st and 2nd antibody, and the blots were visualized by enhanced chemiluminescent (ECL) detection solutions (GE Healthcare, USA).

### Xenograft mouse model experiments

All animal experiments were conducted in accordance with recommendations in the National Research Council’s Guide (IACUC, Republic of Korea) for the Care and Use of Laboratory Animals. The experimental protocol was approved by the Animal Experiments Committee of Duksung Women’s University (permit number: 2014-015-007). Balb/c nude mice (female, 5 weeks old; Joong-ang Animal Experiment Company, Republic of Korea) were used as our xenograft animal model. Mice were housed individually on a 12-h day/12-h night cycle at 23 ~ 27 °C and had access to food and water. Mice were randomly divided into two groups (*n* = 10/group): (1) a drinking water group (*n* = 10): animals received oral administration of drinking water; (2) a CME group (*n* = 10): animals received oral administration of *Cordyceps militaris* ethanol extract (100 mg/kg). To produce tumors, each mouse was implanted with RKO cells (1 × 10^6^ cells per animal), subcutaneously in the back next to the right hind leg. Next, *Cordyceps militaris* ethanol extract (100 mg/kg) or drinking water was administered orally every day for 3 weeks. Fourteen days later, the tumors were identified and then measured every 2 days with a Standard Caliper. Tumor volume was calculated as follows: tumor volume (mm^3^) = [tumor length (mm) × tumor width (mm)^2^]/2. Once tumor volume reached up to 2000 mm^3^, animals were euthanized.

### Statistical analysis

Data were processed using Microexcel software and were expressed as mean ± SD. Comparisons of several means were performed using one-way or two-way analysis of variance followed by the Fisher’s exact test to identify significant differences between groups. P values of less than 0.05 were considered significant.

## Results

### Cytotoxicity of *Cordyceps* extract in RKO cells

**Fig. 1:**
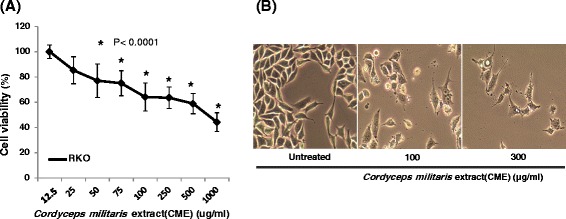
Cell cytotoxicity of *Cordyceps militaris* in human colorectal carcinoma RKO cells. **a** RKO cells were treated with various concentrations (0–1000 μg/mL) of ethanol extract of *Cordyceps militaris* (CME) for 48 h, and cell viability was determined by CCK-8 assay. The results are presented as mean ± standard deviation (SD) for five independent experiments. **b** Cells were treated with or without ethanol extract of *Cordyceps militaris* (CME) for 24 h or 48 h. Cell morphology was observed by light microscopy (×200)

To investigate the cytotoxic effect of ethanol extract of *Cordyceps militaris* on human colorectal carcinoma RKO cells, cells were treated with serially diluted extract of *Cordyceps militaris* (0, 25, 50, 75, 100, 250, 500, and 1000 μg/ml) for 48 h and cytotoxicity was determined by CCK-8. Figure 1a shows that ethanol extract of *Cordyceps militaris* starts to inhibit cell viability at a concentration of 75 μg/ml compared to untreated cells (*P* < 0.0001). In Fig. 1b, cell morphological changes by two different concentrations of ethanol extract of *Cordyceps militaris* (100 and 300 μg/ml) were observed. There were fewer cells in the extract-treated conditions.

### *In vivo* evaluation of anti-cancer effect of *Cordyceps militaris* ethanol extract in a xenograft mouse model

**Fig. 2:**
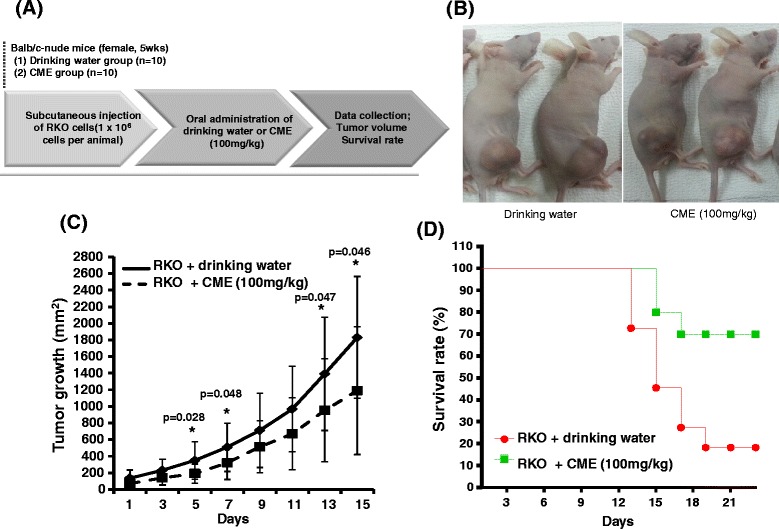
Anti-cancer effect of *Cordyceps militaris* in a xenograft mouse bearing RKO cell–derived human colorectal cancer. Mice were injected with human colorectal carcinoma RKO cells (1 × 10^6^ cells per mouse) subcutaneously into the back next to the right hind leg. Mice were sorted into 2 groups (*n* = 10/group) and orally administered ethanol extract of *Cordyceps militaris* (CME) (100 mg/kg) or drinking water. 14 days later, tumors were identified and measured every two days until the experimental endpoint. **a** Study design for animal experiment. **b** Photograph of xenograft mice bearing RKO cell-derived human colorectal cancer in right hind leg. The pictures of untreated and treated groups were taken at 13 days since the tumor volume was measured. **c** Inhibitory effect of *Cordyceps militaris* (CME) on RKO cell-derived tumor growth. Compared between drinking water group and CME group *P < 0.05. **d** Correlation of survival rate of xenograft mice bearing human colorectal cancer

To evaluate the anti-cancer effect of ethanol extract of *Cordyceps militaris in vivo*, mice were injected subcutaneously with human colorectal cancer RKO cells (1 × 10^6^ cells per mouse) and then ethanol extract of *Cordyceps militaris* (100 mg/kg) or drinking water was orally administrated every day for 3 weeks. Tumor growth was detected from all 20 mice and tumor volume was measured every two days until it reached 2000 mm^3^. Figure 2a displays the overall study design for animal experiment. Figure 2b shows representative photographs of xenograft mice bearing RKO cell-derived human colorectal cancer. The pictures of the extract fed and water fed groups were taken at 13 days since the tumor volume was measured. The size of tumors from the extract fed mice was smaller than that from water fed mice. In Fig. 2c, we confirmed that continuous feeding of ethanol extract of *Cordyceps militaris* (100 mg/kg) significantly inhibited the growth of RKO cell-derived tumors. We further determined whether inhibition of tumor growth directly correlates with survival rate in the xenograft animals. As expected, we observed a reduced mortality in mice administered 100 mg/kg of ethanol extract of *Cordyceps militaris* in Fig. 2d.

### Evaluation of cell cycle arrest in RKO cells treated with *Cordyceps militaris* ethanol extract

**Fig. 3:**
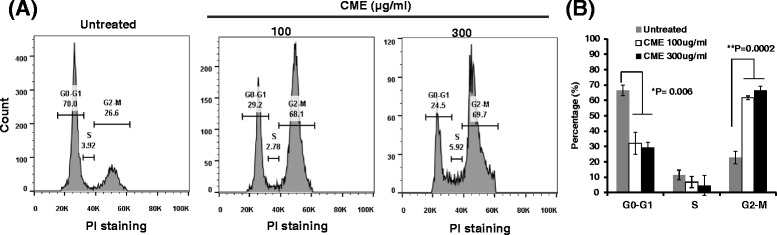
Induction of cell cycle arrest by *Cordyceps militaris* in RKO cells. Cells were treated with or without ethanol extract of *Cordyceps militaris* (CME) (100 and 300 μg/ml) for 24 h. Then, cells were harvested for PI staining, which was measured by flow cytometry. **a** Representative PI staining for cell cycle progress in RKO cells. **b** The percentage of cell cycle distribution is presented as mean ± standard deviation (SD) for five independent experiments. Compared with other groups *P =0.006, **p = 0.0002

To clarify the molecular mechanism of *Cordyceps militaris*, we analyzed the effect on cell cycle regulation in RKO cells using flow cytometry. Interestingly, ethanol extract of *Cordyceps militaris* (100 or 300 μg/ml) induced an increase in the G2/M phase distribution of cells (100 μg/ml; 61.67 %, 300 μg/ml; 66.33 %) as compared to untreated cells (20.5 %) (Fig. 3a). and the quantitative analysis gave a statistically significant difference (Fig. 3b).

### Evaluation of cell apoptosis in RKO cells treated with *Cordyceps militaris* ethanol extract

**Fig. 4:**
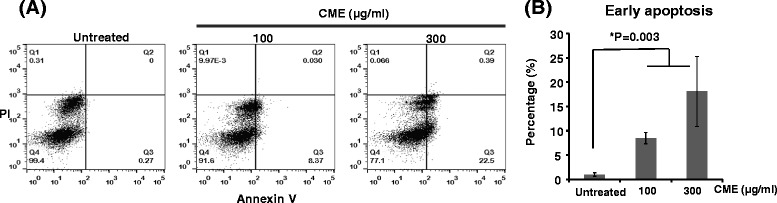
Induction of cell apoptosis by *Cordyceps militaris* in RKO cells. Cells were treated with or without ethanol extract of *Cordyceps militaris* (CME) (100 and 300 μg/mL) for 48 h and then stained with Annexin V and propidium iodide (PI). Apoptosis of RKO cells was analyzed by flow cytometry. **a** Representative Annexin V and PI staining for cell apoptosis in RKO cells. **b** The percentage of early apoptosis is presented as mean ± standard deviation (SD) for five independent experiments. Compared with other groups *P = 0.003

We further examined its effect on cell apoptosis using Annexin V-FITC and propidium iodide (PI) staining. As shown in Fig. 4a, 100 or 300 μg/ml of ethanol extract of *Cordyceps militaris* induced 8.37 % or 22.5 %, respectively, early apoptosis in RKO cells. Five times of independent experiments showed that ethanol extract of *Cordyceps militaris* significantly increased early apoptosis compared to untreated cells(untreated; 1.01 %, 100 μg/ml; 8.48 %, 300 μg/ml; 18.07 %) (Fig. 4b).

### Evaluation of apoptotic-related protein expression in RKO cells treated with *Cordyceps militaris* ethanol extract

To identify the responsible apoptotic-related molecules, we examined the expression of p53, Bcl-2 family proteins, caspases, and poly (ADP-ribose) polymerase (PARP) using western blot analysis. Mitochondria-mediated apoptosis involves a variety of signaling molecules including p53, caspase activators, the proapoptotic and antiapoptotic Bcl-2 family proteins [18–20]. We found increased expression of p53 and proapoptotic Bcl-2 family proteins (Bim, Bax, Bak, and Bad) in RKO cells treated with ethanol extract of *Cordyceps militaris*.

**Fig. 5:**
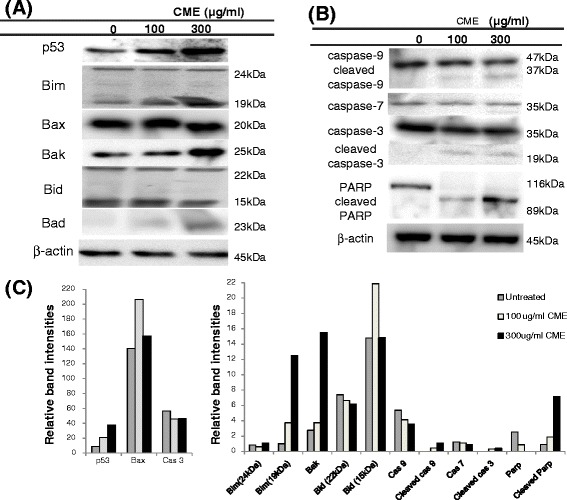
Induction of mitochondrial-mediated apoptosis by *Cordyceps militaris* in a p53-dependent manner. Cells were treated with or without ethanol extract of *Cordyceps militaris* (CME) (0, 100, and 300 μg/ml) for 48 h and then western blot analysis for various apoptotic related molecules were performed. β-actin served as the loading control. **a** Expression of p53 and Bcl-2 family proteins. **b** Expression of caspases 9, 7, and 3, and PARP proteins. **c** The relative intensity of each band compared to the loading control

In addition, the release of cytochrome *c* is known to activate caspase-9 and caspase-3. Activation of caspase 3 triggers the cleavage of PARP (89 and 24 kDa), which functions to prevent DNA damage [25]. As shown in Fig. 5b, the extract of *Cordyceps militaris* increased the cleaved forms of caspases 9 and 3, which are final effector molecules in mitochondrial apoptosis.

## Discussion

In this study, we investigated the anti-cancer effect of ethanol extract of *Cordyceps militaris* in human colorectal carcinoma RKO cells. *Cordyceps militaris* showed potent cell cytotoxicity to RKO cells (Fig. 1), which is consistent with previous studies conducted in other human cancer cell lines [26, 27]. Our *in vitro* cell cytotoxicity was correlated with inhibition of tumor growth of human colorectal cancer observed in a xenograft mouse model (Fig. 2c). A few research groups have studied the anti-cancer effect of *Cordyceps militaris* in a xenografted animal model before. Park *et al.* reported an anti-tumor effect with a water extract of *Cordyceps militaris* in lung cancer cell-derived tumors [28]. The amount of *Cordyceps militaris* extract used in that study was either 150 mg/kg or 300 mg/kg, which is slightly higher than the amount (100 mg/kg) we used in this study. Therefore, we speculate that ethanol extract of *Cordyceps militaris* is more potent than water extract [28].

We also found that the anti-cancer effect of *Cordyceps militaris* in RKO cells was directly associated with induction of cell cycle arrest and apoptosis (Figs. 3 and 4). In fact, Yang *et al.* previously reported that *Cordyceps militaris* arrested human glioblastoma cells in the G0/G1 phase of the cell cycle. Further, Mollah *et al.* reported that *Cordyceps militaris* arrested human colon cancer HT-29 cells in the G2/M phase of the cell cycle, which agrees with our results [26, 29]. Therefore, there may be a differential regulation in cell cycle progression between human glioblastoma cells and human colon-related carcinoma HT-29 or RKO cells. In terms of apoptosis, *Lee et al.* reported that cordycepin, the major ingredient of *Cordyceps militaris*, induced cell apoptosis in HT-29 cells, which is consist with our present study in RKO cells treated with ethanol extract of *Cordyceps militaris* [30]. Also, cordycepin was reported to stimulate Hep3B human hepatocellular carcinoma cells to TRAIL-mediated apoptosis [31]. In addition, Cordycepol C, one of sesquiterpene compounds from Cordyceps extract, showed to cause poly(ADP-ribose)polymerase-1 (PARP-1) cleavage and triggered the loss of mitochondrial membrane potential (ΔΨ_m_) in HepG2 cells in a time- and dose-dependent manner [32].

Finally, we identified that the responsible molecules for cell apoptosis by *Cordyceps militaris* are caspases 3 and 9, Bim, Bax, Bak, and Bad, which are critical components in the mitochondrial pathway of cell apoptosis. This result is consistent with a recent study by Zhang *et al.* [33]. Interestingly, we also found increased expression of cleaved PARP (89 kDa) in RKO cells treated with ethanol extract of *Cordyceps militaris* (Fig. 5b). As mentioned earlier, the activation of caspase-3 is known to trigger the cleavage of PARP, which indicates the halt of PARP function [25]. Therefore, *Cordyceps militaris* induces mitochondria-dependent apoptosis in RKO cells, which involves an increase of pro-apoptotic Bcl-2 family proteins, activation of caspases 9 and 3 and cleavage of PARP.

As mentioned early, traditional herbal medicines have become one of the most important alternative treatments for cancer therapy. These days, publics are also aware of the significance of prophylactic medications to complement or prevent various cancers. Therefore, we consider our ethanol extract of *Cordyceps militaris* could be a good nutritional supplement for cancer therapy. We will further characterize the active compounds from our extract for anti-cancer effect and investigate the responsible molecular mechanisms in detail.

## Conclusions

Ethanol extract of *Cordyceps militaris* inhibited the growth of human colorectal carcinoma RKO cells as well as the *in vivo* growth of tumor in xenograft mice. The inhibitory effect on human colorectal carcinoma cells was highly connected with cell cycle arrest and p53-dependent, mitochondrial mediated apoptosis.

## Abbreviations

CME: Extract of *Cordyceps militaris*
ATCC: American type culture collection
ECL: Enhanced chemiluminescene
FITC: Fluorescein isothiocyanate
